# Biased phylodynamic inferences from analysing clusters of viral sequences

**DOI:** 10.1093/ve/vex020

**Published:** 2017-08-03

**Authors:** Bethany L. Dearlove, Fei Xiang, Simon D. W. Frost

**Affiliations:** 1 Department of Veterinary Medicine, University of Cambridge, Madingley Road, Cambridge, CB3 0ES, UK

**Keywords:** phylodynamics, genetic clustering, transmission, molecular epidemiology, infectious diseases

## Abstract

Phylogenetic methods are being increasingly used to help understand the transmission dynamics of measurably evolving viruses, including HIV. Clusters of highly similar sequences are often observed, which appear to follow a ‘power law’ behaviour, with a small number of very large clusters. These clusters may help to identify subpopulations in an epidemic, and inform where intervention strategies should be implemented. However, clustering of samples does not necessarily imply the presence of a subpopulation with high transmission rates, as groups of closely related viruses can also occur due to non-epidemiological effects such as over-sampling. It is important to ensure that observed phylogenetic clustering reflects true heterogeneity in the transmitting population, and is not being driven by non-epidemiological effects. We qualify the effect of using a falsely identified ‘transmission cluster’ of sequences to estimate phylodynamic parameters including the effective population size and exponential growth rate under several demographic scenarios. Our simulation studies show that taking the maximum size cluster to re-estimate parameters from trees simulated under a randomly mixing, constant population size coalescent process systematically underestimates the overall effective population size. In addition, the transmission cluster wrongly resembles an exponential or logistic growth model 99% of the time. We also illustrate the consequences of false clusters in exponentially growing coalescent and birth-death trees, where again, the growth rate is skewed upwards. This has clear implications for identifying clusters in large viral databases, where a false cluster could result in wasted intervention resources.

## 1. Introduction

Boosted by the increasing use of viral genotyping for clinical purposes, there are more HIV sequence data than ever, representing around a third of all viral sequences in Genbank, which can also be used to characterise the transmission dynamics of HIV. Grouping sequences into phylogenetic clusters has previously proven useful for sifting through these large datasets, with particular focus on national-level databases, and this approach has been used to correlate transmission with contact rates, social network structures, risk behaviours, and the presence of co-infections with other viruses in a number of HIV studies, including those from the United Kingdom, Switzerland, Canada, the Netherlands and South America (Hughes et al. 2009; Bezemer et al. 2010; Kouyos et al. 2010; Ragonnet-Cronin et al. 2010; Junqueira et al. 2016). However, the definition of a phylogenetic cluster has not so far been standardised, despite there being numerous approaches and software implementations to identify them (Prosperi et al. 2011; Ragonnet-Cronin et al. 2013; Dennis et al. 2014; Wertheim et al., 2014; Vrbik et al. 2015).

In standard epidemiology, a cluster is defined as a higher than expected burden or incidence of disease in close proximity in time and space (Porta 2008). In phylogenetics, a cluster is simply a group of closely related sequences, or a subtree within the full phylogeny, linked by a single recent common ancestor. This definition on its own does not help resolve epidemiological links, since broad clustering in a tree of all HIV-1 sequences in a national database will simply identify subtypes (Wertheim et al. 2014). However, this does not mean to say that the phylogenetic and epidemiological definitions of a cluster cannot be reconciled; sequences that are more closely related genetically may have a smaller distance in terms of the number of transmission events that have occurred between them, all other things being equal. Thus, the identification of phylogenetic clusters can represent groups of recent transmission when identified using a measure of relatedness. Whilst exact definitions vary in the literature, this relatedness, referred to here as the threshold, is usually in the form of an allowable distance between sequences, either through a cut-off of genetic diversity, or in a time period reflective of time to diagnosis. Methods using the mean, median, and maximum distance for the threshold have been proposed (Hughes et al. 2009; Leigh Brown et al. 2011; Prosperi et al. 2011). Constraints on the certainty of the subtree (through a bootstrap or posterior probability, for example), subtree size, and geography of isolates have also been introduced (Hué et al. 2005; Kouyos et al. 2010).

**Figure 1.**
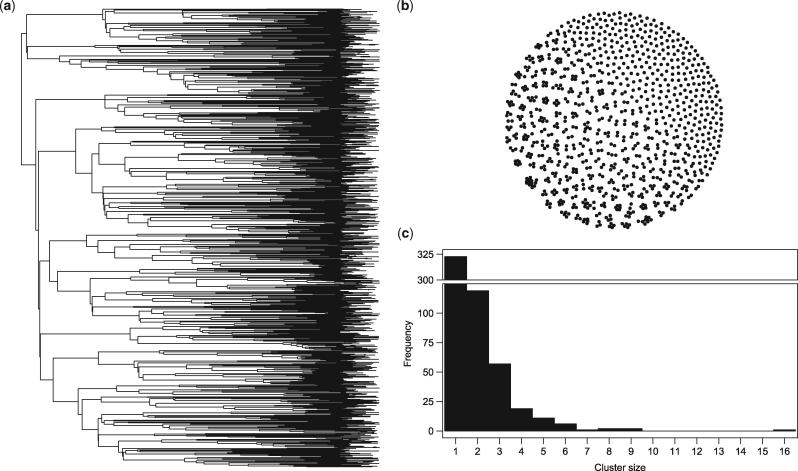
Identifying clusters using a threshold. Clusters from a simulated HIV phylogenetic tree [Village 0 from Ratmann et al. (2017)] permuted to remove any substructure (a) with a threshold cut-off of 15 years (Dearlove and Frost 2015) can also be represented by an undirected network (b) or size (component) distribution (c).

Regardless of how clusters are identified, resulting clusters are undirected between pairs of sequences, and thus can also be drawn as a network (Fig. 1b). The sizes of clusters can be represented as a (component) distribution (Fig. 1c). The distribution of cluster sizes is often found to be right skewed, with a large number of very small clusters and fewer large ones, shown to be well-fitted by a power-law distribution (Hughes et al. 2009; Leigh Brown et al. 2011). This shape is often thought to be a reflection of the heterogeneity in transmission, particularly for sexually transmitted infections where a similar pattern of skew can be seen in risky sexual contacts (Liljeros et al. 2001). Cluster sizes that are unusually large and fall in the upper tail of the size distribution, such as the cluster of size 16 in Fig. 1, tend to be of particular interest since these suggest new or unexpected patterns of transmission for further analysis. However, without a null model in which there is no heterogeneity in transmission, it is difficult to interpret these distributions. Indeed, there is nothing special about the largest cluster in Fig. 1, since the phylogeny has been randomly permuted to remove any substructure (Dearlove and Frost 2015; Ratmann et al. 2017).

The main advantage of clustering sequences from a large database is that it is fast and scalable (Ragonnet-Cronin et al. 2013), helping to downsize large sequence databases to groups of particular interest for further in-depth analyses that would be computationally infeasible on the database as a whole. When we have meta-data about clustered individuals, such as potential risk behaviours and clinical results, then clusters can help identify trends between individuals linked within the same cluster and elucidate where to target interventions. Interest also lies in whether these clusters may represent distinct sub-epidemics associated with higher transmission rates. If this were the case, then clusters can be treated separately from the dataset as a whole. However, clusters of related sequences can arise by chance even in the absence of any transmission heterogeneities in the dataset, simply due to the asymmetric way that random trees branch, and can also be driven by oversampling. In this case, treating a cluster of sequences as distinct may be misleading.

Biases can also arise regardless of whether or not clustering is present. Kuhner et al. (1998) have previously showed that estimates of the growth rate in coalescent models of exponential growth are biased upwards. Two factors they attributed this to were the non-linearity of the relationship between the growth rate and the coalescence times, and the censoring of the first coalescence event due to sampling. Pybus et al. (2000) and Wiuf (2003) showed that the amount of bias varies according to the product of the effective population size and the growth rate. Censoring has also been found to be an issue in non-parametric estimates of the effective population size over time, with false slowing of epidemics detected near the present (de Silva et al. 2012).

In this paper, we investigate the problems of using phylogenetic clusters for phylodynamic inference, motivated by the large national database scenario. These national-level phylogenies are a composite of the local transmission process and higher level migration events, including from multiple subtypes. Whilst each sub-epidemic might resemble a typical HIV-1 phylogeny with long branches need the tips, the overall tree can have multiple, complex topological features to tease apart. Identifying a sub-epidemic with different transmission dynamics relative to the rest of the epidemic early in its development is essential for intervention to have the greatest impact. One way to do this is to consider estimates of epidemiological parameters in transmission clusters to provide insights into the ancestral dynamics (Dennis et al. 2014), and identify whether clusters truly represent sub-epidemics with different transmission patterns. Here we focus on the issues of false clusters, that is, clusters identified using the threshold method but from phylogenies simulated under models that are randomly mixing and therefore unstructured. We investigate two main modelling frameworks, the coalescent and birth–death sampling process, quantifying parameter estimates of the effective population size and growth rate of an epidemic. For the coalescent models, we also implement a model that incorporates censoring for the first coalescence event.

## 2. Methods

### 2.1 Simulating phylogenies

Coalescent phylogenies were simulated in GENIE v3.0 (Pybus and Rambaut 2002), with the population size in the present, *N(0)* equal to 1,000 generations, and the exponential growth rate, r, equal to 0.5. Trees with 200, 400, 600, and 800 tips were simulated under each scenario, with 1,000 replicates at each sample size. A further 500 phylogenies were simulated with 500 tips, *N(0)* of 1,000 generations and *r* equal to 0.01, 0.05, 0.1, 0.5, 1.0, and 5.0 to test the effect of varying the growth.

Birth–death trajectories were simulated using MASTER v 4.1.3 (Vaughan and Drummond 2013), with a birth rate of 0.2 and death rate of 0.1 per unit of time, and an initial population size of one. We again simulated 1,000 phylogenies of 200, 400, 600, and 800 tips, with samples taken homochronously after 70 time units. Therefore, in contrast to the coalescent case, more tips represent a higher sampling proportion rather than total population size. We removed any simulations that failed to reach the target sample size.

### 2.2 HIV-like trees

To better understand the problems of clustering on a more realistic HIV-like phylogeny, we consider village-level trees simulated and made available as part of the Phylogenetics And Networks for Generalized HIV Epidemics in Africa (PANGEA-HIV) methods comparison exercise (Ratmann et al. 2017). These simulations cover a localised population size of around 8,000 individuals, with 25–50% sequencing coverage depending on the scenario. To create a null distribution of trees with no population substructure, we used the permutation method described in Dearlove and Frost (2015) to simulate 500 new trees from each of Village simulations 0, 6, and 7. This method retains the tip and internal node times from the observed phylogeny, but reconnects them at random, therefore breaking down any epidemiological effects. This means that any clusters subsequently identified will be false positives.

### 2.3 Cluster identification

The phylogeny was first converted into a matrix of pairwise distances between tips. Samples were deemed to be in the same cluster if the total branch length, or patristic distance (Farris 1967), between them was less than the threshold distance. The exact threshold depended on the modelling scenario, and was chosen to ensure that the largest cluster used in all further analyses had enough tips to provide useful estimates, but did not include the full tree. Therefore, the star-like trees of the growing populations required thresholds relatively deeper in the phylogeny so as to maintain largest clusters that were not just singletons and pairs. In the constant coalescent simulations we used a threshold of 500 time units, with 15 in the exponential case when *r = *0.5. For the extra exponential phylogenies with 500 tips, thresholds were chosen so as to produce similar distributions for the maximum cluster size (Supplementary Fig. S1). In the birth–death scenario, we used a threshold of 50 time units and for the HIV-1 PANGEA trees, we used a threshold of 15 years.

To assess the impact of the threshold value, we also looked at two other scenarios. To investigate the effect of conditioning on the largest cluster size at a particular threshold distance, we chose an internal node at random, and all tips linked by that as their most recent common ancestor were deemed to be in the cluster. Internal nodes were resampled if the resulting cluster had less than four tips. This is denoted the ‘random subtree’ method in what follows. We also dropped tips at random throughout the tree (the ‘randomly dropping tips’ method), leaving the same size tree as the largest cluster, to assess any bias in estimates that would be expected from simply having a smaller phylogeny.

### 2.4 Model fitting and parameter estimation

All models were fitted using maximum likelihood in R (R Core Team 2013). For the coalescent simulations, we fitted the constant ($N\left(t\right)=N(0)$), exponential ($N\left(t\right)=N\left(0\right)e^{-rt}$) and logistic ($N\left(t\right)=N(0)(\alpha +\left(1-\alpha \right)e^{-rt})$) demographic coalescent models, where *N(0)* is the initial population size, r is the exponential growth rate and α is the population size at t=∞ as a proportion of $N\left(0\right)$. Optimization was performed via the BOBYQA algorithm (25) as implemented in the minqa package in R (Bates et al. 2014) using the genieR package available on GitHub (available at: https://github.com/xiangfstats/GenieR). The model with the best fit was decided by taking the model with minimum Akaike Information criterion (AIC) (Akaike 1974).

For the birth–death model, we followed the fitting procedures given in Section 3 of Volz and Frost (2014). The likelihood was maximised using the Nelder–Mead method (Nelder and Mead, 1964) implemented in the bbmle package in R (Bolker 2016). We assume the death rate is known and fixed at 0.1, and estimate the birth rates and sampling proportion. This makes sense epidemiologically, since these are often parameters of interest during outbreak situations, and independent clinical information can be used for the death rate.

The percentage change in parameter estimate is calculated as:

$$\frac{\theta_{small}-\theta_{full}}{\theta_{full}}\times 100$$

where $\theta_{full}$ is the estimate in the full phylogeny, and $\theta_{small}$ is the estimate from the down sampled tree (i.e. identified cluster, randomly sampled subtree or from randomly dropping tips). When this value is negative, the smaller tree underestimates the true parameter, and when it is positive, the smaller tree overestimates the full tree parameter.

### 2.5 Censored likelihood

To investigate the effect of left censoring of the first coalescent event, we implemented a censored likelihood. Only the first coalescent event needs to be adjusted for censoring, since the exponential waiting time in the coalescent model is calculated between consecutive internal nodes in the tree. Therefore, for the second and deeper coalescence events, we can calculate the exact waiting times from the tree. However, for the first coalescence event, the observed time is cut off by the sampling of the lineages below it. Under the standard coalescent (Kingman 1982a,b), the probability density function for the time to the next coalescence is:

$$p\left(t\right)=\left(\begin{array}{c} \text{k}\\ 2 \end{array}\right)\lambda \left(t\right)\exp\;\left(-\left(\begin{array}{c} k\\ 2 \end{array}\right)\Lambda \left(t\right)\right)$$

where *k* is the number of lineages, $\Lambda \left(t\right)=\int_{0}^{t}\lambda \left(u\right)du$, and $\lambda \left(t\right)$ is the rate of coalescence relative to that in the present (Griffiths and Tavaré 1994). Therefore, the probability that the first coalescence event has a time, $C_{1}$, greater than the observed $t_{1}$, the time of sampling, is given by:

$$P\left(C_{1}>t_{1}\right)=1-P\left(C_{1}\leq t_{1}\right)=\exp\;\left(-\left(\begin{array}{c} k\\ 2 \end{array}\right)\Lambda \left(t_{1}\right)\right).$$

The total likelihood is given by the product of all the probabilities of the coalescence events and sampling events, that is:

$$\prod_{i}p(t_{i})$$

where

$$p\left(t_{i}\right)=\left\{\begin{array}{cc} \exp\;\left(-\left(\begin{array}{c} k\\ 2 \end{array}\right)\Lambda \left(t_{i}\right)\right) & \text{if}\;i\;\text{is}\;\text{the}\;\text{first}\;\text{coalescence}\;\text{or}\;a\;\text{sampling}\;\text{event}\\ \left(\begin{array}{c} k\\ 2 \end{array}\right)\lambda \left(t_{i}\right)\;\exp\;\left(-\left(\begin{array}{c} k\\ 2 \end{array}\right)\Lambda \left(t_{i}\right)\right) & \text{if}\;i\;\text{is}\;\text{the}\;\text{second}\;\text{or}\;\text{later}\;\text{coalescence}\; \end{array}\right.$$

Thus, the censored likelihood is different from the standard coalescent likelihood by a factor of $\left(\begin{array}{c} k\\ 2 \end{array}\right)\lambda \left(t_{1}\right)$.

## 4. Results

### 4.1 Constant coalescent model

We considered coalescent phylogenies simulated under two demographic scenarios: constant and exponential growth.

**Figure 2.**
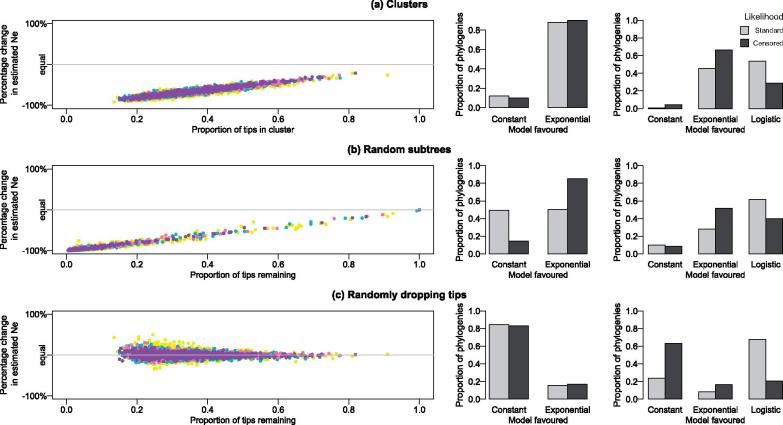
The effect on estimates of the population size in a constant coalescent when considering (a) clusters, (b) random subtrees, and (c) tips dropped at random. Colours represent the number of tips in the tree: yellow = 200, green = 400, pink = 600, and purple = 800. The bar plots show the proportion of phylogenies favouring the constant versus exponential (middle column) or constant versus exponential and logistic models (rightmost column) for the standard (light grey) and censored (dark grey) likelihoods.

In the constant coalescent, the estimated effective population size of the full population was underestimated when considering the false-positive clusters (Fig. 2). The effect was similar whether the false-positive cluster was found using a threshold, shown in Fig. 2a, or using a random subtree (Fig. 2b). The bias was strongest when considering clusters containing the smallest proportion of the full tree, reducing as the cluster size approached that of the full tree. This makes sense, since in a coalescent model, the effective population size is proportional to the branch lengths in the tree. By analysing the cluster without the remaining ancestral context, the depth of the tree has been lost. When trees are down-sampled to the same number of tips at random, then the full depth of the tree is less likely to be lost and the estimate of the full tree more likely to be recovered (Fig. 2c). What is perhaps more surprising is the linear nature of the relationship between proportion of tips in the cluster or subtree, and the reduction in estimate of the full effective population size (Table 1). For every 10% (i.e. a proportion of 0.1) more tips present in the cluster relative to the full tree, the accuracy of the effective population size increases by around 9% in the constant case, and this is consistent regardless the number of tips in the tree.

**Table 1.** Regression estimates and 95% confidence intervals for the constant coalescent simulations in the form of *y* = *β*_0_* + β*_1_*x*, where *y* is the percentage change in estimated *Ne*, and *x* is the proportion of tips in the cluster.

Clustering approach	Intercept, *β*_0_	Slope, *β*_1_	Adjusted *R*-squared
Threshold cluster	−100.14 (−100.55, −99.73)	92.00 (90.84, 93.15)	0.8591
Random subtree	−100.43 (−100.47, −100.39)	97.80 (97.37, 98.23)	0.9805
Random tips	4.52 (3.70, 5.34)	−11.16 (−13.47, −8.86)	0.02181

Although of theoretical interest, the effective population size of a cluster relative to the full tree is less useful in practice. The effective population size is hard to interpret in absolute terms with respect to the number of infected individuals, as it is confounded by the transmission rate (Frost and Volz 2010, 2013); however, exponential growth rates as well as overall demographic patterns are well captured when sampling is random. Therefore, we also performed model fitting to the false clusters, to see if they most resembled a constant, exponential or logistic population.

Surprisingly, 67.8% of true constant trees were wrongly fitted as having logistic growth when dropping tips at random, which should have little or no effect on the demographic model (Table 2). When we instead did model fitting using the censored likelihood, this figure was reduced to 20.4% (constant: 63.2%; exponential: 16.4%). Using clusters biased the model selection further: when comparing between the constant and exponential, the constant model was favoured only 12.0% of the time for clusters compared to 84.6% for trees with randomly dropped tips (censored likelihood: 10.0% versus 83.2%). Adding the logistic model caused the constant model to be favoured for only 1% of the clusters, versus 23.9% of trees with randomly dropped tips (censored likelihood: 4.6% versus 63.2%).

**Table 2.** Model fitting results.

	Percentage best fitted by the AIC using:					
	Standard coalescent			Censored coalescent		
Model	Constant	Exponential	Logistic	Constant	Exponential	Logistic
*Constant*						
Threshold cluster	0.650	45.525	53.825	4.475	66.600	28.825
Random subtree	10.100	28.325	61.575	8.600	51.575	39.825
Random tips	23.900	8.350	67.750	63.175	16.400	20.425
Full tree	24.950	8.130	66.920	69.375	15.300	15.325
*Exponential*						
Threshold cluster	0.000	52.150	47.850	0.000	58.750	41.250
Random subtree	10.950	51.800	37.250	0.000	61.650	38.350
Random tips	0.000	90.700	9.300	0.000	95.050	4.950
Full tree	0.000	90.725	9.275	0.000	92.350	7.650

This is particularly problematic when it comes to phylodynamic inference for identifying intervention strategies. Using a metapopulation coalescent (Dearlove and Wilson 2013), it can be shown that exponential growth of the viral population is equivalent to a susceptible-infectious (SI) epidemiological model in the host, and the logistic curve is equivalent to the susceptible-infectious-susceptible model (SIS).

### 4.2 Exponential coalescent model

**Figure 3.**
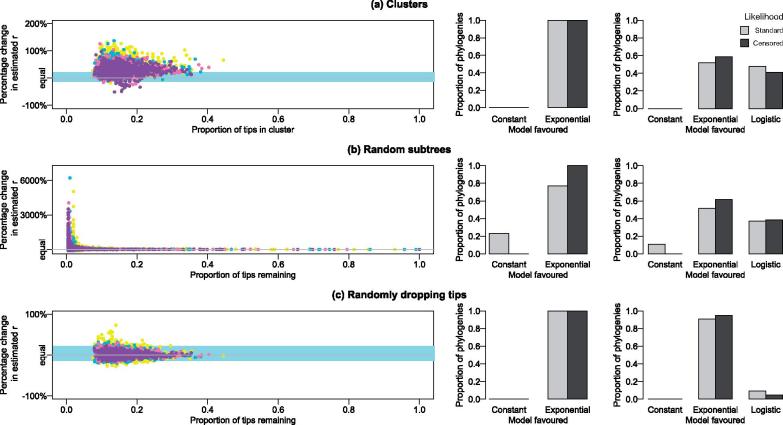
The effect on estimates of the growth parameter, *r*, in an exponential coalescent when considering (a) clusters, (b) random subtrees, and (c) tips dropped at random. Colours represent the number of tips in the tree: yellow = 200, green = 400, pink = 600, and purple = 800. The blue region shows the 2.5 and 97.5% points of the bias in the randomly dropped tips trees for comparison. The bar plots show the proportion of phylogenies favouring the constant versus exponential (middle column) or constant versus exponential and logistic models (rightmost column) for the standard (light grey) and censored (dark grey) likelihoods.

The results for the effective population size of the exponential model were broadly similar to that of the constant model (Supplementary Fig. S2), though with much more noise. The long branch lengths in the present relative to the past, typical of exponential tree shape, meant that the effective population size tended to be overestimated when using particularly small random subtrees, and also when tips were randomly dropped. The constant demographic model was rejected for all cases (Fig. 3), with the exponential model rejected for 47.9% of the clusters but only 9.3% of the trees with randomly dropped tips (censored likelihood: clusters 41.3% versus dropped tips 5.0%).

For growing populations, using false clusters might seem less problematic than in the simplest constant case if there was other evidence, such as epidemiological data, also suggesting a growing population. However, Fig. 3 shows that the estimates of the growth parameter, *r*, tend to be biased upwards in clusters relative to the true value in the tree. This overestimation is beyond that which occurs when tips are dropped at random (Fig. 3c). The bias calculated from these randomly dropped tip trees represents the expected bias simply due to having a smaller tree, and has a median value of 1.19%, with 2.5 and 97.5 percentiles of −13.74 and 22.03%, respectively. For comparison purposes, these extremes are represented by the light blue panels in Fig. 3a,b.

**Figure 4.**
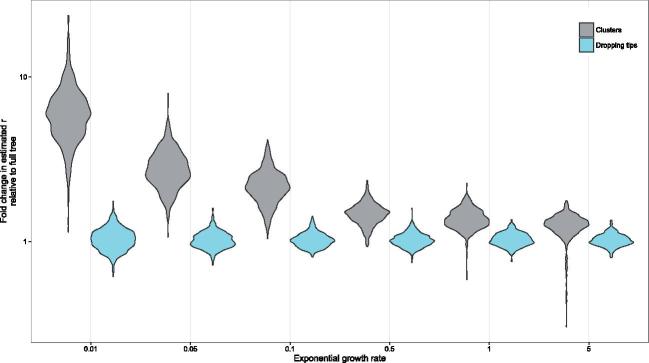
Comparison of fold change in estimated *r* for downsampled trees relative to the full tree estimate for clusters (grey) and dropping tips (blue). Thresholds were chosen to obtain similar maximum cluster sizes across the different values of *r* (shown in Supplementary Fig. S1).

When taking clusters in these trees (Fig. 3a), the median growth parameter bias is 33.27%, with 94.52% clusters having more extreme bias than the same size tree obtained by dropping tips at random. More generally, points are estimated to fall above light blue box 73.58% percent of the time. A slightly lower figure is obtained for the random subtrees (63.1%), where the smaller subtrees give much more varied estimates overall. The pattern of overestimation is much more pronounced for smaller exponential growth rates, with 99.6% of clusters falling outside of the 2.5–97.5% interval from randomly dropping tips when *r *= 0.01, but less so for larger ones, for example only 66.0% of clusters fall outside the equivalent interval for *r* = 5 (Fig. 4).

### 4.3 HIV-like phylogenies

**Figure 5.**
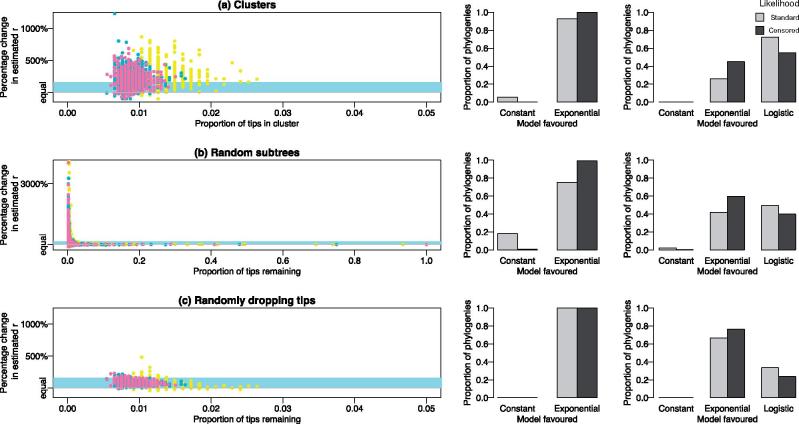
The effect on estimates of the growth parameter, *r*, in the HIV-like phylogenies when considering (a) clusters, (b) random subtrees, and (c) tips dropped at random. Colours represent the PANGEA Village Simulation number: yellow = 0, green = 6, and pink = 7. The blue region shows the 2.5 and 97.5% points of the bias in the randomly dropped tips trees for comparison. The bar plots show the proportion of phylogenies favouring the constant versus exponential (middle column) or constant versus exponential and logistic models (rightmost column) for the standard (light grey) and censored (dark grey) likelihoods.

We considered three datasets from the PANGEA methods comparison exercise, Villages 0 (low percentage acute, no intervention), 6 (high fraction acute, fast intervention scale up), and 7 (high fraction acute, slow intervention scale up). We took 500 permutations of each, fitting an exponential coalescent as the full model. All the villages showed similar levels of bias (Fig. 5). As would be expected from the exponential simulations, the estimated growth rates in the subtrees were biased upwards. For subtrees with randomly dropped tips, the median bias is 68.00%, with 2.5 and 97.5 percentiles of −3.86 and 161.07%, respectively. For clusters, the median bias is 217% (−20.34%, 545.70%), with 87.67% clusters having more extreme bias than their equivalent dropped tips tree.

**Figure 6.**
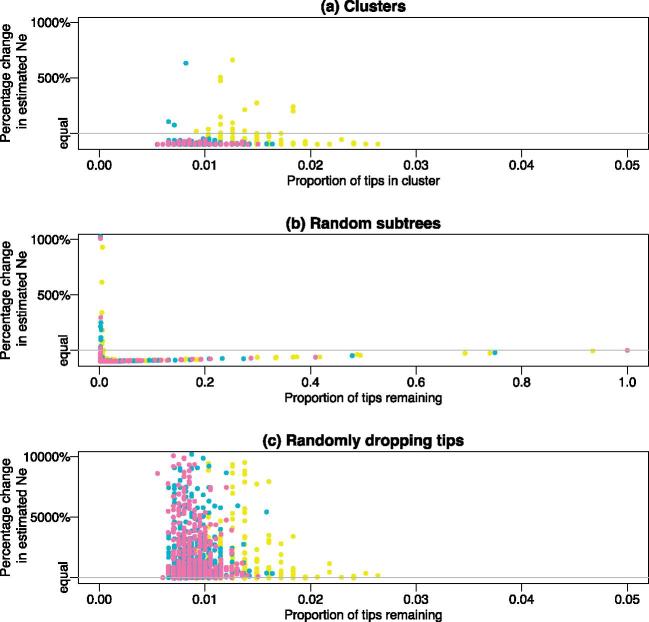
The effect on estimates of the present population size, *N*_0_, in the HIV-like phylogenies when considering (a) clusters, (b) random subtrees and (c) tips dropped at random. Colours represent the PANGEA Village Simulation number: yellow = 0, green = 6, and pink = 7.

The results for the effective population size were much more variable (Fig. 6). Whilst the bias for the clusters in general followed the trend seen in the constant and exponential coalescent models, with biases closer to −100% the fewer the tips remaining in the tree, the bias for the randomly dropped tip trees were regularly overestimated. For these trees, the median bias was 700%, whereas it would be expected to centre around zero. This most likely reflects the small size of the trees with variable heights depending on the sampling dates, and also some model mis-specification. Similar results were obtained assuming the logistic model as the true full model.

### 4.4 Birth–death models

The birth–death results look very similar to those of the exponential coalescent. Like *r*, the exponential growth parameter, the birth rate, *b*, was overestimated in the clusters (Supplementary Fig. S3). When randomly dropping tips, the median overestimate is 1.84%, with the 2.5 and 97.5 percentiles −13.38% and 42.92% respectively (again, represented by the light blue panels in Supplementary Fig. S3). The median overestimate rises to 22% (−23.33, 457.90) for the randomly chosen clades, and 31.5% (0.69, 94.22) using threshold clustering. Making a direct comparison between the dropped tips and cluster results, since they have the same down sampled size, the cluster result bias is more extreme (whether over or underestimating) than of the dropped tip tree in 90.8% of the phylogenies.

The population size, calculated using the size of the tree and the estimated sampling proportion, is also underestimated (Supplementary Fig. S4). The line on the graphs shows where *y = x*, and when dropping tips at random, the full tree estimate is recovered. Clusters and random subtrees underestimate the majority of the time, with results generally noisier in the latter: the cluster overestimates the population for 1.4% of phylogenies, versus 5% for random clades and 57.95% when dropping tips at random. Similar results were obtained when using the tree likelihood conditioned on the number of tips in the phylogeny [as given by Equation 3 in Stadler (2013)].

## 5. Discussion

Early identification of new epidemics is essential for interventions to be most effective. This is even more important in a highly clustered epidemic, where, unless interventions are targeted appropriately, the infection will continue to spread regardless of its underlying transmissibility (Leigh Brown et al. 2011). In this paper, we show that whilst clusters can be useful for finding affinities between closely related samples, there are some caveats when using them to understand demographics in subpopulations. Most importantly, false clusters can masquerade as growing epidemics.

Clusters have previously been used in the literature to downsample large datasets and look at the fit of demographic models (Hué et al. 2005; Mir et al. 2016; Patiño-Galindo et al. 2016). For example, Hué et al. (2005) identified six clusters of UK origin, and found all of them to be best fitted by a logistic growth model with an doubling time of approximately a year during the initial exponential growth stage. Although their clusters were identified using location, rather than genetic or temporal signal, the results of our random subtree analysis (where we could have labelled tips at random with location and picked the biggest cluster with >90% identical geography) show that it is feasible that these clusters are not growing at all, and are simply an artefact of using a threshold to downsample a large database taken from a randomly mixing population.

Identifying clusters is not the only problem when inferring population dynamics, however. Previously noted biases were reiterated when looking at our trees downsampled by randomly dropping tips: the over-estimation of the exponential growth rate parameter and the false fitting of the logistic model over the constant (Kuhner et al. 1998; Pybus et al. 2000; Wiuf 2003; de Silva et al. 2012). de Silva et al. (2012) showed that there exist biases in non-parametric estimates of the population size, with false slowing of the exponential growth parameter near to the present. In their paper, they suggested that this is due to left censoring backwards in time and the relative time between the first coalescence and sampling events. Here, the majority of true constant downsampled trees were wrongly fitted as having logistic growth when using standard parametric models. We implemented a censored likelihood for the first coalescent event which went some way to improving the model selection, suggesting that demographic biases from censoring are not limited to non-parametric methods (c.f. the conclusions of de Silva et al.). The censoring appeared to have less effect on the model selection in clusters, probably due to the overall reduced tree height.

Clustering itself has a number of disadvantages, particularly pertaining to the arbitrary choice of threshold. Most clustering methods are defined by non-parametric methods, that is, not by a model, and neglect the rest of the information deeper in the phylogeny’s ancestry, with small thresholds biasing cluster membership to recent infection events. Non-epidemiological factors such as sampling are also an issue (Frost and Pillay 2015); oversampling in an area or time period can skew results, and unsampled individuals mean that a point source transmission to a single cluster cannot be excluded.

When clusters truly represent different subpopulations, it makes sense computationally to analyse them outside the background of the rest of the database. One obvious problem is that large databases will not have one single background demographic model as considered here. Instead, there may be one or more clusters of varying age, demography and viral viability. Effect on demography estimates aside, the phylogenetic clustering is ill-equipped for dealing with these. A recent simulation study by Poon (2016) showed that many clustering methods cannot detect heterogeneity in transmission rates between subpopulations. Instead, they focus on individuals with a short waiting period between transmission and diagnosis. This has further ramifications on targeting interventions, as clusters may well be identifying subpopulations that are already seeking medical attention.

Methods for identifying up-and-coming epidemics from within a large database need to be able to look for growth above and beyond the biases we have shown exist. Clustering approaches have the advantage that they are reasonably fast even on relatively large datasets (Ragonnet-Cronin et al. 2013), especially compared to alternative approaches such as the structured coalescent. To truly identify clusters, it seems likely that an explicit structured coalescent or similar is required, where clusters can be determined parametrically. There have been a number of recent developments which have made the structured coalescent more amenable for this sort of analysis, however fitting many demographic models to an unknown number of stratifications in the sample remains difficult (Volz et al. 2012; Rasmussen et al. 2014; De Maio et al. 2015). One way to understand the population background in the tree for comparison to an identified cluster would be to use information in the clades outside of the cluster to form a null distribution. The clustering method of Prosperi et al. (2011) uses genetic distance between tips for a similar purpose. For phylodynamic inference, a permutation approach such as that in Dearlove and Frost (2015) could be used to assess whether observed clustering is due hidden heterogeneity. In addition to asymmetry metrics for measuring heterogeneity across the phylogeny, the clade size distribution, local branching index (Neher et al. 2014), and exponential growth or birth rate calculated from different time points (e.g. for the subtree below each internal node) could add additional information. However, statistical power is likely to become an issue, as the overall phylogeny could contain many conflicting local transmission dynamics which might be affecting its topology.

Until a suitable null distribution or parametric method for identifying clusters is developed, their interpretation will remain problematic for phylodynamics. In particular, we should be cautious when targeting intervention strategies on the basis of clustering, to ensure resources are put where they will have most impact, and not to populations already active in seeking medical help.

## Supplementary Material

Supplementary Data

## Data Availability

The simulated phylogenies and R code for analyses are available on github at: https://github.com/bdearlove/Papers/tree/master/ClusteringBias. **Conflict of interest:** None declared.
