# Supplementary material for: Biased phylodynamic inferences from analysing clusters of viral sequences
Source: Virus Evol. 2017 Aug 3;3(2):vex020. doi: 10.1093/ve/vex020 (PMC5570026; doi:10.1093/ve/vex020)
Supplement: Supplementary Data [file vex020_supp.pdf]

Biased phylodynamic inferences from analysing clusters of viral sequences

Bethany L. Dearlove[1], Fei Xiang[1], Simon D. W. Frost[1]

[1]Department of Veterinary Medicine, University of Cambridge, Cambridge, United Kingdom

Supplementary Information

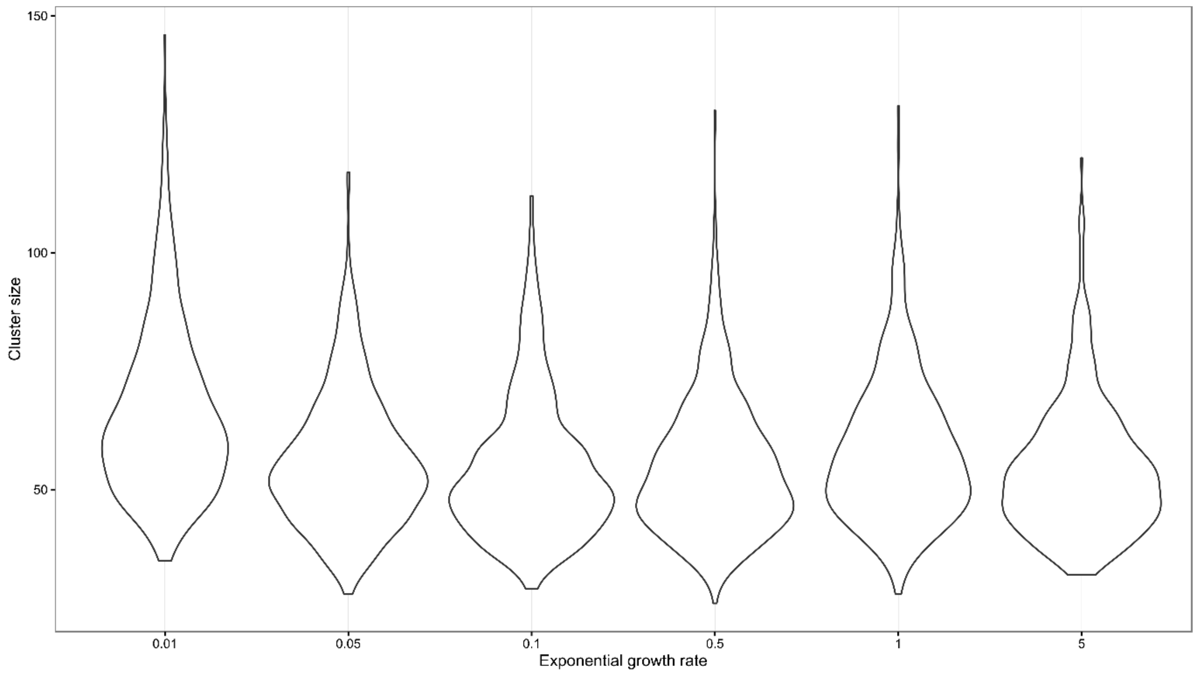

Supplementary Figure 1. Violin plots showing the maximum cluster sizes used when comparing between different exponential growth rates.

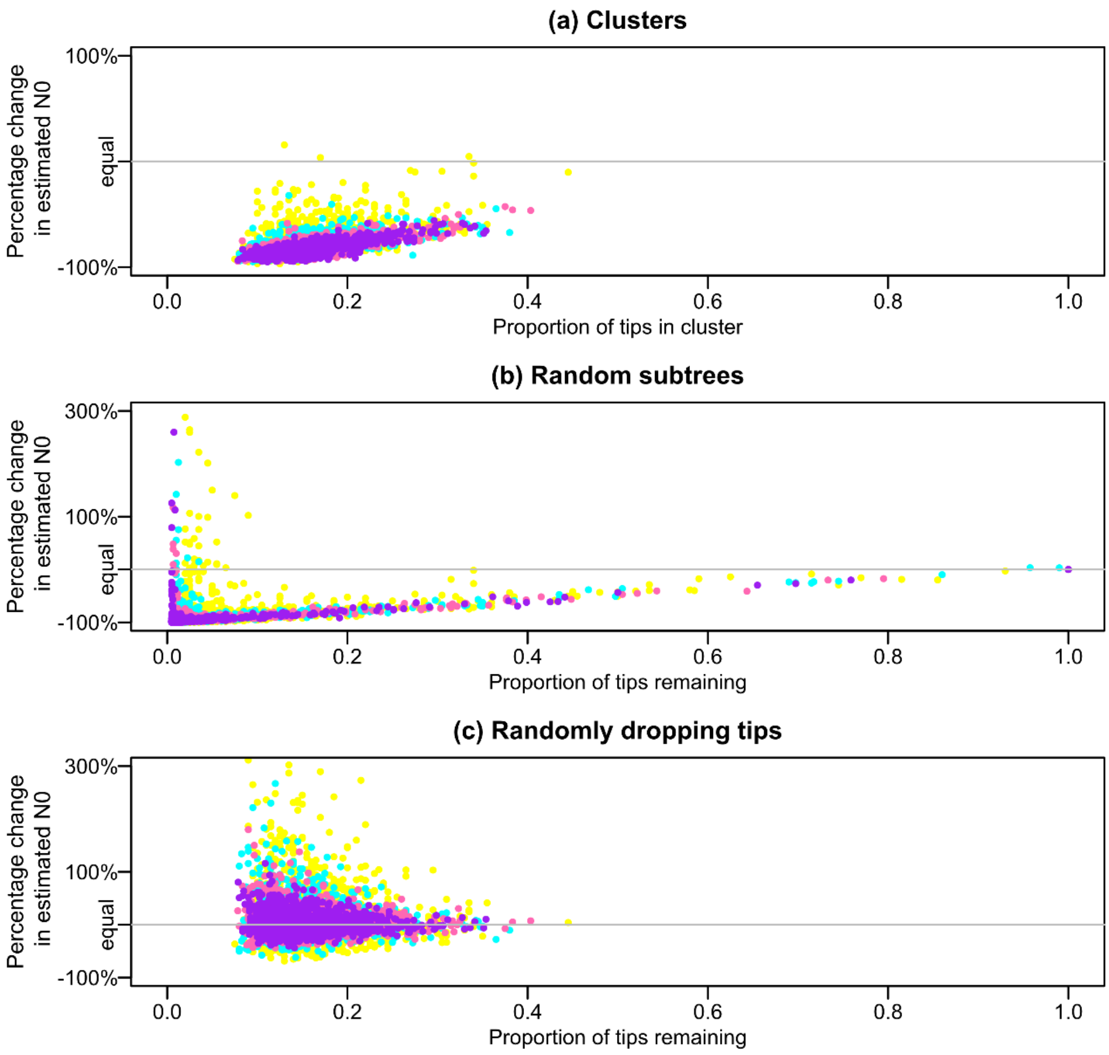

Supplementary Figure 2. The effect on estimates of the present population size, N_0, in an exponential coalescent when considering (a) clusters, (b) random subtrees and (c) tips dropped at random.

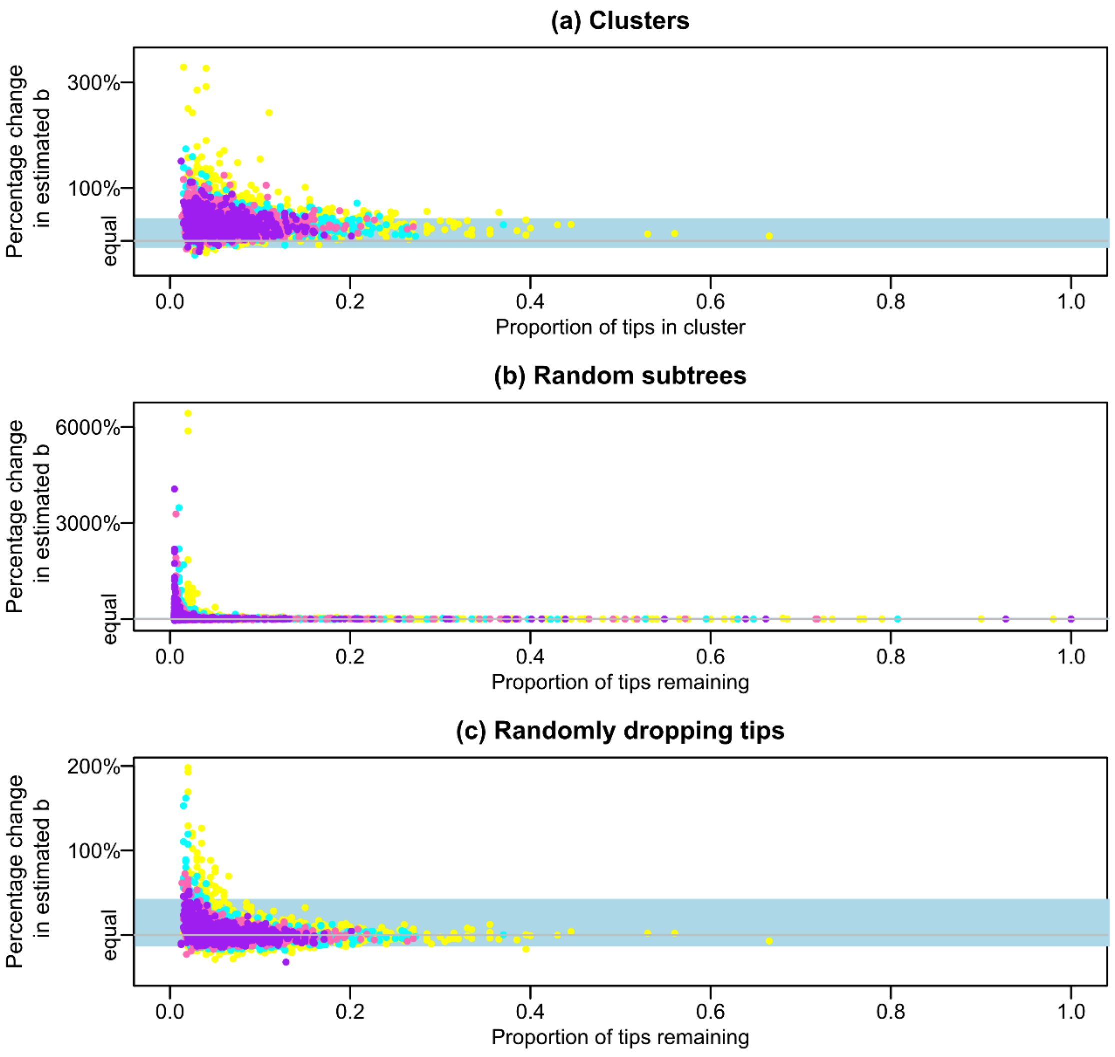

Supplementary Figure 3. The effect on estimates of the birth rate, *b*, from the birth-death model when considering (a) clusters, (b) random subtrees and (c) tips dropped at random. Colours represent the number of tips in the tree: yellow = 200, green = 400, pink = 600 and purple = 800.

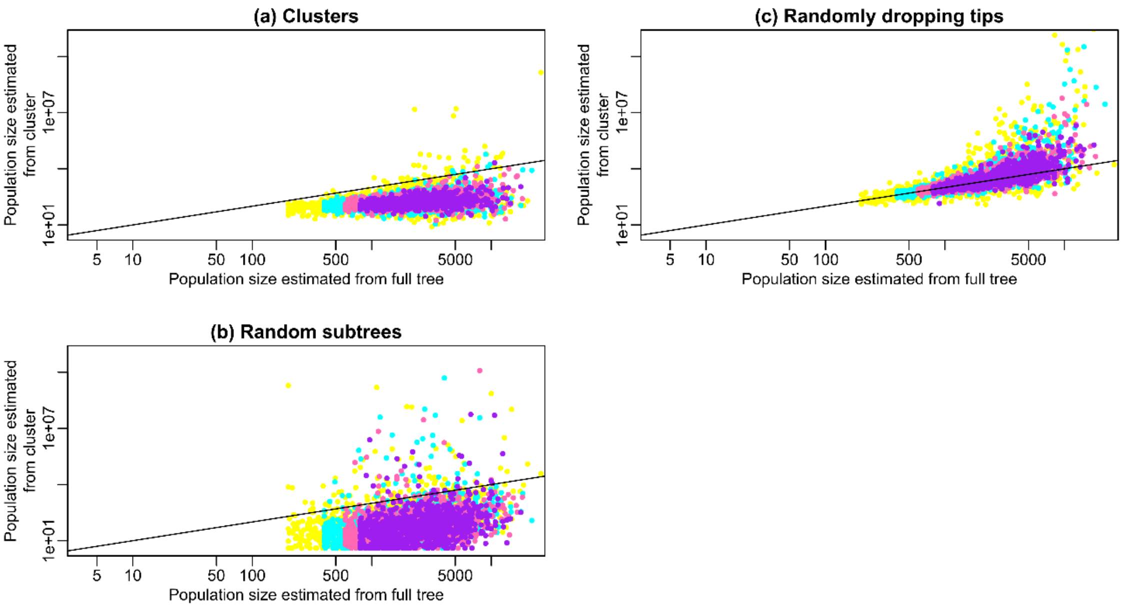

Supplementary Figure 4. The effect on estimates of the population size as estimated from the sampling proportion and tree size in from the birth-death model when considering (a) clusters, (b) random subtrees and (c) tips dropped at random. The line shows where $y = x$. Colours represent the number of tips in the tree: yellow = 200, green = 400, pink = 600 and purple = 800.
